# A Visual Attentive Model for Discovering Patterns in Eye-Tracking Data—A Proposal in Cultural Heritage

**DOI:** 10.3390/s20072101

**Published:** 2020-04-08

**Authors:** Roberto Pierdicca, Marina Paolanti, Ramona Quattrini, Marco Mameli, Emanuele Frontoni

**Affiliations:** 1 Dipartimento di Ingegneria Civile, Edile e dell’Architettura, Universitá Politecnica delle Marche, 60131 Ancona, Italy; r.quattrini@univpm.it; 2 Dipartimento di Ingegneria dell’Informazione, Universitá Politecnica delle Marche, 60131 Ancona, Italy; m.paolanti@pm.univpm.it (M.P.); m.mameli@pm.univpm.it (M.M.); e.frontoni@univpm.it (E.F.)

**Keywords:** eye-tracking, Digital Cultural Heritage, Deep Convolutional Neural Networks

## Abstract

In the Cultural Heritage (CH) context, art galleries and museums employ technology devices to enhance and personalise the museum visit experience. However, the most challenging aspect is to determine what the visitor is interested in. In this work, a novel Visual Attentive Model (VAM) has been proposed that is learned from eye tracking data. In particular, eye-tracking data of adults and children observing five paintings with similar characteristics have been collected. The images are selected by CH experts and are—the three “Ideal Cities” (Urbino, Baltimore and Berlin), the Inlaid chest in the National Gallery of Marche and Wooden panel in the “Studiolo del Duca” with Marche view. These pictures have been recognized by experts as having analogous features thus providing coherent visual stimuli. Our proposed method combines a new coordinates representation from eye sequences by using Geometric Algebra with a deep learning model for automated recognition (to identify, differentiate, or authenticate individuals) of people by the attention focus of distinctive eye movement patterns. The experiments were conducted by comparing five Deep Convolutional Neural Networks (DCNNs), yield high accuracy (more than 80%), demonstrating the effectiveness and suitability of the proposed approach in identifying adults and children as museums’ visitors.

## 1. Introduction

Over time, art galleries and museums have conserved our important Cultural Heritage (CH) and many museums and exhibitions employ technologies to enhance the museum visit experience. With the increasing spread of devices for human behavior analysis, many systems have been developed to increase and personalise the visitors’ experience. Currently, visitors have an active role within museums, as they are the main agents in increasing their knowledge and learning. Researchers’ efforts have been devoted to a better description and comprehension of the motivations of why people do or do not visit museums, what they do there, and what learning/meaning they derive from the museum experience. Nowadays, it is more and more important to develop strategies enabling insiders to tailor the CH offerings according to the users’ needs and preferences [1]. These visits are by now recognized as an important potential for people to make up and explore their own mentality, and to check their own perception in a place of the experts [2,3]. Furthermore, research is focused on user transformation as an actor of the procedures for the acquisition of raw data [4].

Art is perceived in subjective manner by people. Their reactions can be different, due to the narrative of the art piece, for religious content or even for the various techniques used to represent it. In some paintings, interest can be focused on the non-figurative elements such as lines, colours, textures, and shapes, and in others on the interactions between subjects. However, for many paintings, studies report similar feelings, thus proving that there is coherence in the interpretation of colours and patterns when observing these paintings [5].

This fact is due to the Human Visual System (HVS), which can promptly identify the most important and informative portions of a scene for further analysis. This ability allows us to direct limited processing sources to the most significant visual input [6]. Furthermore, visual cognition science demonstrates that humans, when observing a scene without a specific task to perform, do not focus on each area of the image with the same intensity. Instead, attentive mechanisms guide their gazes to salient and relevant parts [7]. Many existing visual attention models use a set of linear filters, for example, Gabor or center-surround (DoG) filters as a front-end, the outputs of which are non-linearly combined into a real number that indicates visual saliency [8]. Despite these models being able to measure the location salience, they are of finite use, because the generated salience maps do not always match actual human fixations, and the performance of the models largely depends on tuning many parameters [9]. Since gaze fixations match with the true human visual attention, an encouraging way to enhance the visual attention model is to use the eye tracking data. This methodology gives the position of the eye for determining the gaze direction of a person at a given time and also the sequence in which they have been moved [10]. The use of such technology can be considered as a key to address users’ expectations and needs, to assess the intrinsic value of CH resources and to collect usage statistics by the users. Traditional eye-trajectories-based action recognition methods mainly focus on designing hand-crafted features to model the temporal pattern of coordinates sequences. Conversely, with the increasing development of deep learning technologies, Deep Convolutional Neural Networks (DCNNs) have been exploited to model the temporal dynamics of trajectories sequences in References [11,12]. Moreover, Convolutional Neural Networks (CNNs) based methods convert sequences to color images and then adopt CNNs to learn the discriminative spatio-temporal characteristics, which have achieved superior performances [13]. In this paper, we have proposed a novel Visual Attentive Model (VAM) that is learned from eye tracking data. As demonstrated by our previous work in this field [14,15], the exploitation of a user’s gaze proved to be particularly useful for the development of Augmented Reality applications [16]. In fact, while the current practice for the implementation of such kinds of multimedia experience is to deliver applications from the domain expert’s point of view, it has been proved that a user-centered design is more effective, with the contents tailored according to the user’s preferences. With reference to the previous study, we have increased the dataset by collecting eye-tracking data of adults and children observing five paintings with similar characteristics. The images are selected by CH experts and are—the three “Ideal Cities” (Urbino, Baltimore and Berlin), the Inlaid chest in the National Gallery of Marche and Wooden panel in the “Studiolo del Duca” with Marche view. These pictures have been recognized by experts in the domain with analogous features thus providing coherent visual stimuli. Besides this, our presented method combines a new coordinates representation from eye sequences by using Geometric Algebra with the deep learning model for the automated recognition (to identify, differentiate, or authenticate individuals) of people by the attention focus of distinctive eye movement patterns. We firstly construct an eye-sequence space as a subset of Geometric Algebra to represent the eye coordinates. Then a view transformation is proposed to overcome the diversity of viewpoints.

The main considerations that motivated our solution are as follows—(1) There are strong spatial correlations between different coordinates in each eye trajectory, so that the coordinates contain discriminative representations of the eye attention focus. (2) Temporal dynamics of the eye sequence can be explicitly described as the relative motion between adjacent coordinates. In short, the coordinates representations of the eye sequence are the direct embodiment of the spatial and temporal characteristics respectively, which dictate how the eye moves in practice. Based on these obtained images, five state-of-the-art classifiers, namely VGG-16 [17], VGG-19 [17], Inception-ResNet [18], GoogLeNet [19] and ResNet-50 [20], are compared to recognize the type of user observing the paintings.

The paper is organized as follows—Section 2 gives a brief look at the latest achievements in the analysis of eye-tracking data, specifically for the CH domain. Section 3 gives details the collection of the data acquired and the paintings selection for our analysis (Section 3.1) and it introduces our approach consisting of representation transformation (Section 3.2), and the design of the DCNN models for user classification; (Section 3.3). Section 4 describes the results. Conclusions and future works are presented in Section 5.

## 2. Related Works

The exploitation of eye-tracking data, as is well known, has proven to be valuable in many domains such as food [21], neuromarketing [22,23] and many more. Its benefits lie in the detection of the eye’s movements, which can be collected when users interact with computer interfaces. In the literature protocols and methods have been developed that are worth mentioning. In Reference [24], the authors developed methods for analyzing gaze data collected with eye tracking devices. In the paper, they described how to integrate it with object recognition algorithms. The authors of Reference [25] proposed a method for the automatic analysis of mobile eye-tracking data in natural environments and for processing this data by applying face, object, and person detection algorithms. Other studies deepen the bulk of knowledge for the treatment of gaze data. In particular, Nakano and Ishii [26] have examined the use of eye gaze as a marker of user engagement. They have also tried to adjust it to individual users. An index of interest can be the engagement. Pfeiffer et al. [27] have described the EyeSee3D method. In order to align virtual proxies with real-world objects, the authors have combined modeling with 3D marker tracking. In this way, the object fixations can be automatically classified, while supporting an absolutely free moving participant. When they evaluated the pose estimation accuracy, they have inferred that the marker detection failed when the involved subjects observed sideways and there was no marker visible, or simply the head moves quickly or the position changes are extreme. Further, in Reference [28], Ohm et al. performed a user study and assessed object visual attraction with the eye tracker. The authors tried to determine the observed area in a large-scale indoor environment and the objects that can help the subjects in finding their way. The results have shown that functional landmarks such as doors and stairs are the areas in which the attention is most focused and named as landmarks. Videos have been studied as well, like in the work of Ma et al. [29], which argued about the capability to extract user models based on users’ eye gaze observing videos. Given the above, it can be stated that some studies proposing physical markers have been attached to each area of interest (AOI) for identifying them, while others, for the detection of many objects, report large scale variations entailed in a lower detection rate. Referring to our experiments, we decided to collect data without AOI constrains, supported by our previous research [15].

Obviously, such methods are fundamental to uncover users’ behavioural patterns when dealing with multimedia. The research community need a baseline to understand the benefits of learning and usability. Lai et al. [30] have proposed a literature review about the use of eye tracking technology in learning studies. In this paper, the authors classified the studies considering the different areas of cognitive development by using eye tracking technology and the connections established between eye movement measures and learning purposes. This work has proven that perception, conceptual development and language were the mainly studied areas of cognitive development. In Reference [31], eye-tracking technology was adopted to explore cognitive activities in multimedia learning. The authors describe that by using eye movement measurements it is possible to provide the opportunity to test assumptions about where people look during the integration of text and pictures. In Reference [32], the authors performed a study to show how eye tracking can be used to gain additional insights into how participants work with case study materials. They introduced different variables for analyzing case materials such as fixation duration, dwell time, or reading depth. education. In References [33,34], Schrammel et al. examined user attentional behavior on the move. The challenges and potential of using eye tracking in mobile applications are discussed, demonstrating the ability to use it to inspect the attention on advertising media in two situations. A general attention model based on eye gaze was proposed. The use of an eye-tracking retrospective think-aloud for usability evaluation and description of this application in assessing the usability of a mobile health app is assessed in Reference [35]. The authors used an eye-tracking retrospective think-aloud to evaluate the usability of an HIV prevention mobile app among 20 young men (15–18 years) in New York City, NY; Birmingham, AL; and Chicago, IL.

Back to the specific topic of our study, even Cultural Heritage can benefit from eye-tracking studies [36]. In Reference [37], eye movement was evaluated by a spatio-temporal point process model for comparing the eye movements of experienced and inexperienced art viewers. However, the model developed by the authors is not accurate for studying the complete dynamics of the fixation process, since it does not capture how the fixation process changes over time. In Reference [38], the authors studied how to identify user attention by eye tracking in the tourism domain, by investigating tourist boredom observing a city view. In Reference [39], the authors studied the statistical regularities in artwork related to low-level processing strategies in human vision, especially for the processing of natural scenes and the variations in these statistics. Quiroga et al. [40] have deduced that people observed an artwork considering the subject’s interests and their artistic appreciation. This assumption derived from the pattern of fixations of subjects looking at figurative and abstract paintings from various artists and at modified versions and several aspects of these art pieces were changed with digital manipulations. The influence of bottom-up and top-down processes on visual behaviour of subjects, while they observe representational paintings is the main goal of the work proposed in Reference [36].

Although technology is mature enough to provide reliable and accurate data, research efforts are still needed for the post-processing phase. In other words, findings about behavioural patterns are entrusted to a standard classification approach. Given the recent advances in Artificial Intelligence, it is the time to experiment with novel ways of data processing. Some recent works move toward this direction. In Reference [41], a machine learning method for facial stimuli for emotion recognition is proposed. The authors have recorded the eye-movements of subjects with autism that performed three facial analysis tasks—free-viewing, emotion recognition, and brow-mouth width comparison. The authors of Reference [42] described the learner’s visual attention distribution and to what extent visual attention patterns in instructor-present videos predict learning and learner perceptions. The eye movements of the participants have been simultaneously registered using a desktop-mounted eye tracker. In Reference [7], Cornia et al. presented a model which can predict accurate saliency maps by incorporating neural attentive mechanisms. Their solution is based on a convolutional long short-term memory that in the input image draws attention to the regions most salient for iteratively refining the predicted saliency map. Besides, their model can learn a set of prior maps generated with Gaussian functions.

To infer and describe the user observing similar paintings, and considering the great effectiveness of DCNNs to model the temporal dynamics of trajectories sequences, we have proposed a novel VAM, which combines the eye sequences with a deep learning approach. This provides new answers to predict the museums’ visitor characteristics. The main contributions of this paper, aside from extending the system and the analysis presented in References [14,15], are (i) the proposal of a novel VAM to help identify the type of user in front of paintings; (ii) the collection and analysis of a real eye-tracking CH dataset for deep learning purposes that is public to all researchers and also useful to art researchers and artists alike (http://vrai.dii.univpm.it/content/eye-tracking-dataset-ch); (iii) an in-depth analysis to investigate whether there is a positive attentional bias when looking at similar paintings, and (iv) a real environment test with results that prove the main goals of the paper mentioned above.

## 3. Material and Methods

**Figure 1:**
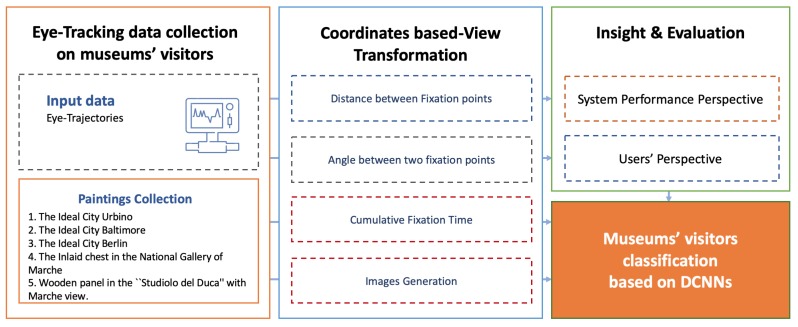
Overview of our Visual Attentive Model (VAM) for museums’ visitors classification.

The approach presented in Reference [15] has been extended for the development of the proposed VAM. The framework is schematically depicted in Figure 1 and comprises three main components—the eye-tracking data acquisition, the Coordinates-Based View Transformation, the museums’ visitors classification. Further details are given in the following subsections. The framework is comprehensively evaluated on the five paintings with similar characteristics chosen by CH experts domain. The details of the data collection are discussed in Section 3.1.

### 3.1. Setup and Acquisition

The eye-tracking data are recorded by using a Tobii Eye-Tracker X2-60 and the Imotions®Attention Tool software (vers. 5.7), as described in Reference [14,15]. The eye-tracking dataset stores eye-tracking data collected from 34 participants to the tests. In particular, the 18 adults taken in exam in this work were Italian students and employees at Universitá Politecnica delle Marche. Instead, the 16 children were students of a primary school. As already stated, the main goal of this research is to evaluate the differences of adults and children while observing paintings. The user behaviour understanding is really important to enhance and personalise the museum visit experience. We started by selecting two classes of users regardless of their background and knowledge of the CH field. In particular, the class of adults comprises some experts of art and the others have experiences by deepening their knowledge of various artistic processes related to secondary school studies. The children participants were used to attending museums on school trips, but none of these were experts in the CH domain. Furthermore, the participants involved in this work had never used or had never seen the eye-tracking system before this study. Such differentiation in sample of panelists does not affect, significantly, their behaviours, as demonstrated by our previous research [15]. In both cases, all the acquisitions were collected in a quiet room and under standard illumination conditions. Each participant was seated 60 cm from the eye-tracker and monitor. The dataset collected the values of *FixationX* and *FixationY*, which represented the pixels where the attention of the user is focused. Furthermore, we recorded the *FixationDuration*, that is, the duration in milliseconds of the attention at the point of coordinates (FixationX, FixationY).

**Figure 2:**
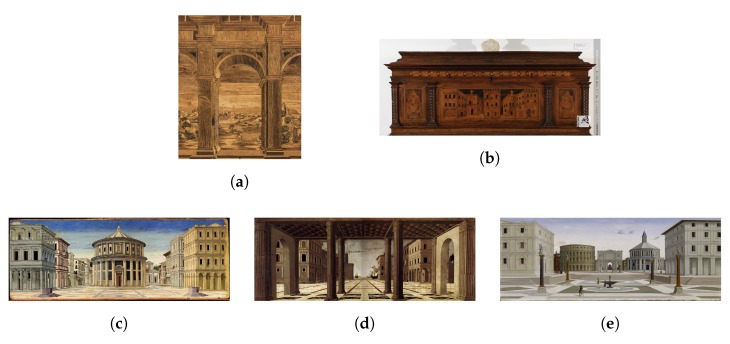
The paintings in our dataset. (**a**) depicts the Wooden panel in the “Studiolo del Duca” with Marche view. (**b**) represents the Inlaid chest in the National Gallery of Marche. (**c**) is the Ideal City in Urbino, (**d**) represents the Ideal City in Berlino, and (**e**) is a picture with the Ideal City in Baltimore.

Figure 2 represents the images selected for the test. These pictures present similar features and were chosen by CH experts in order to provide coherent visual stimuli. From the historiographic point of view, all the pictures are related to the “Ideal City” theme, which is a very large and recurrent topic in the Renaissance age. Those representations are the most beguiling images of a community governed by mathematical order, according to the cosmology and philosophical reflections by the Umanesimo. The paintings in our dataset were the three Ideal Cities (Urbino, Baltimore and Berlin)—they look very similar with differences such as the human presence or the horizon level (Figure 2c–e). It is possible to discover here the concept of Copia et Varietas by Leon Battista Alberti. They all want to act as the definitive vision of the idealized concept of an ideal city, clearly representing the culture of their time. In particular, the painting conserved in the National Gallery of Marche highlights its resemblance to the Ducal Palace, designed as an Ideal city. The most closely dated parallels are some doors, decorated with inlaid wooden panels of ideal cities, in the Ducal Palace at Urbino, which were installed between 1474 and 1482 [43]. For this reason, the eye-tracking data were collected during free viewing in front of the chosen paintings. This setup choice is primarily due because AOIs could influence the eye movements [14]. Instead, our aim is to provide a model that can be as close as possible to a user’s behaviour in museums or art galleries. According to their historical connections, we choose to add in the data set the doors and a wooden panel with a glimpse of a perspective of a landscape from the “Studiolo of the Duke” (Figure 2a,b). They are very different from the paintings with regard to the colors and may seem too uniform. But this fact was intended also as a challenge in the observation training. We had the chance, in this case, to exploit some acquisitions already carried out in the CIVITAS framework, an interdisciplinary research project founded by the Polytechnic University of Marche that aimed to develop and test new paradigms of digitization and fruition for CH in the museum context.

As described in References [14,15], the digital versions of the paintings were shown on a 23${}^{\prime \prime }$ inch monitor at a resolution of 1920×1080 pixels, preserving the original aspect ratio. The eye-movement indicators, on which the analysis is based, are fixations and saccades. Fixations are eye pauses over a particular of interest averaging about 300 ms. Saccades are rapid eye-movements between fixations. Participants were informed that their eye-movements were recorded. The average time of observation registered was 75 s, 15 s for each image. We performed a pre-test, useful for comparing the outcomes between using a digital image and using the real-size artwork.

**Figure 3:**
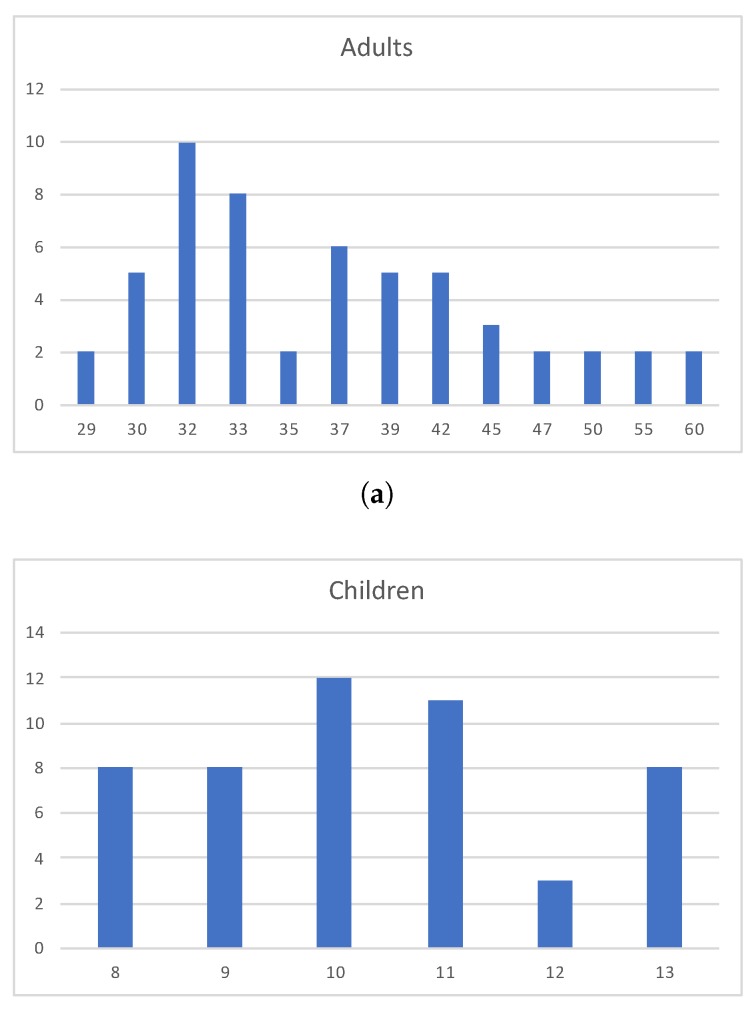
The graphs in this figure represent the age of the people that are involved in the dataset collection. On the horizontal axis there are the ages of the people and on the vertical axis there are the numbers of the people for each age. (**a**) reports the statistics for Adults, (**b**) for children.

The dataset contains data from 105 people: 55 adults and 50 children. The age of adults is in the range from 29 to 60 years. The children were aged from 8 to 13 years. Figure 3 depicts some statistics about the age of the users that constitute our eye-tracking dataset.

### 3.2. Coordinates Based-View Transformation

As stated in the previous Section, we have collected the *FixationX* and *FixationY* coordinates, which represent the geometrical path followed by the users. In particular, thanks to the software *Tobii Studio*, the collection of the data from this device is simplified. At the end of the acquisition the data are exported in the CSV file format with the columns listed and described in Table 1.

Starting from this structure, it is possible to use the coordinates of fixation as the coordinates of a pixel in a new image. Each coordinate of fixation (FixationX and FixationY), represents a pixel where the people focus their attention. For this reason, we have decided to use this information as a position of the pixel in the new images generated. In particular, we have generated 3 images. The first image, namely *Punctual Information (PI)*, is the information that is referred to at the very last stage of the analysis. It is composed by:

- the angle θ of the arrival in that point;
- the Euclidean distance from the previous point;
- the fixation time in the point.

The second image, that we have defined *Mean Information (MI)*, is composed by:

- the distance of the point from the 2 points before that we have called $r_{-2}$;
- the distance of the point from the 3 points before that we called $r_{-3}$;
- the sum of the fixation duration of the 3 previous point.

The third image, called *Long Information (LI)*, composed by:

- the distance of the point from the 5 points before that we have called $r_{-5}$;
- the distance of the point from the 10 points before that we have called $r_{-10}$;
- the sum of the fixation duration of the 10 previous point.

**Figure 4:**
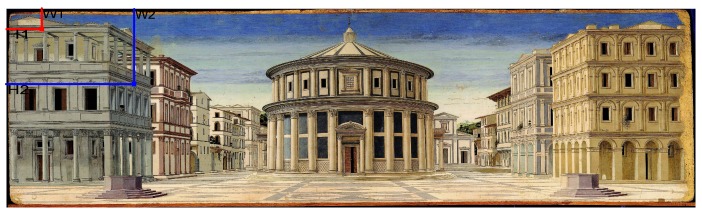
An example of the pixel consideration. Here the pixel dimension is increased for a better understanding.

The angle θ is obtained with the use of the coordinates of the pixels where the user look. In particular, considering the pixels $P_{1}$ and $P_{2}$ in the image with coordinates $\left(W_{1},H_{1}\right)\;\left(W_{2},H_{2}\right)$, expressed by distance from left top corner of the image, the angle is calculated with the Equation (1). An example of the starting point for the angle calculation is showed in Figure 4.

The choice of considering values of 2, 3, 5, 10 as preceding points distance is due to the fact that a point is reachable with different trajectories. For this reason in the *PI* image the first parameter is the θ that permits information about the directionality of the last trajectory used to reach that point.

We have introduced the value $r_{*}$, which represents the Euclidean distance from the considered point. This value is considered the radius of the circle centred in the considered point, for this reason it is reachable from multiple points in the border of this circle. In this way, we have considered that a point is the end of multiple trajectories and we save this information. The information of the distance is combined with the time elapsed to reach the point, as in Equation (2)

(1) $$\theta =arctan\left(\frac{H_{2}-H_{1}}{W_{2}-W_{1}}\right)$$

(2) $$LeadTime=\sum_{i=1}^{n}FixationDuration_{i}$$

where *n* is 3 and 10.

Then, to obtain the information necessary for image creation we have performed a data normalization. In this way, the maximum value in the pixel of coordinates (FixationsX,FixationY) is 255, this is important because the DCNNs used in the next step is based on unsigned 8 bit integer images. The normalization is done by applying Equation (3)

(3) $$NData=\frac{Data*NormalizationConstant}{MaxData},$$

where NormalizationConstant=255 and NData is the result of the normalization and MaxData is the maximum value for the data that is in normalization phase.

We have normalized the obtained value, with a normalization technique based on a simple proportional ratio, for realizing the input images for classification. After this, we have generated a base image and we have inserted the data punctually to each pixel.

### 3.3. Deep Convolutional Neural Networks for Museums’ Visitors Classification

The users’ classification model provides information about the characteristics of a visitor in front of a painting. For this purpose, it is trained with image labels that indicate the users’ class—adults and children. The training is performed by fine-tuning a DCNN. Different DCNNs were tested to choose the ones with the best performance—VGG-16 [17], VGG-19 [17], Inception-ResNet [18], GoogleNet [19] and ResNet-50 [20]. The DCNNs chosen need a huge amount of images to be trained, for this reason the fine-tuning technique is adopted to obtain accurate results. In particular, the training is performed by fine-tuning the networks evaluated that have been pre-trained on the ImageNet dataset to classify images into 1000 categories [44]. Since, this dataset contains over 14 million of pictures divided into 1000 classes, we fine-tune by cutting off the final classification layer and replacing it with a fully connected layer with 2 outputs (one for each class Adult and Children). Moreover, the networks are designed as two blocks, where the first one is the features extraction block and the second one is the classification block, for the first block, the features extraction block is pre-trained on ImageNet and then this block replaces the classification block, which is a fully connected layer, with a block that has an output number based on the number of classes used, in this case two classes.

The learning rate multipliers are increased for that layer. Loss and accuracy layers are adapted to take the input from the newly created final layer. The networks train require 100 epochs with an sgd optimizer and learningrate= 0.0001.

## 4. Results and Discussions

**Figure 5:**
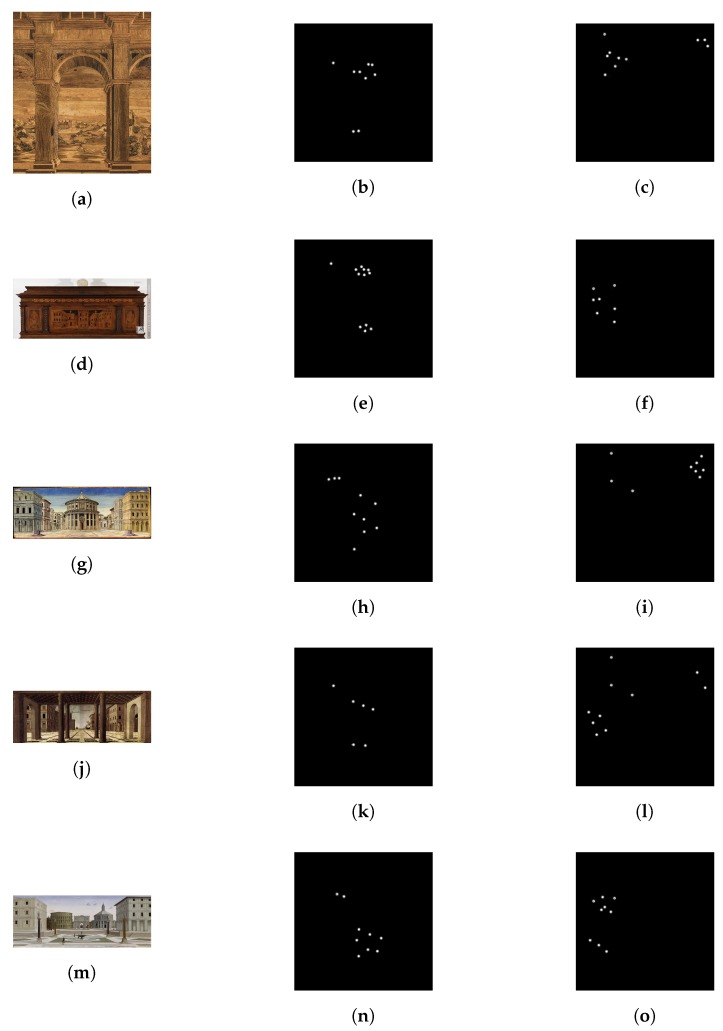
Resulting images after coordinates view transformation. The first column shows the stimulus images; the second column depicts an example of the images generated for the Adult class; the third column reports an example image generated after the coordinates view transformation for the children class.

This paper focused specifically on an analysis of paintings for visitor classification. In this section, the results of our VAM are evaluated. The experiments are based only on these images of the dataset, where at each coordinate (FixationX, FixationY) the values explained before are inserted, as shown in Figure 5. In particular, Figure 5 shows the results of the coordinates view transformation, in which the first column represents the stimulus images, the second column is the fixation point of adult users and the third column depicts the attention of children.

These images reveal that the adult’s attention focuses on the center of the paintings; instead the children’s trajectories are more confused, going from the right to the left side of the images.

These images are all generated with the resolution of 224×224 that is the resolution used by the DCNNs. In particular, after the coordinates transformation, 240 images are obtained for the children’s dataset and 270 images for the adult’s dataset. For the adults and children classification, VGG-16 [17], VGG-19 [17], Inception-ResNet [18], GoogLeNet [19] and ResNet-50 [20] were used and applied to the whole image, trained by fine-tuning a model pre-trained on the ImageNet dataset. We performed the experiments by splitting the labeled dataset into a training set and a test set. The dataset is split into 80% training and 20% test images.

Table 2 presents precision, recall and F1-score of museums’ visitors classification.

**Figure 6:**
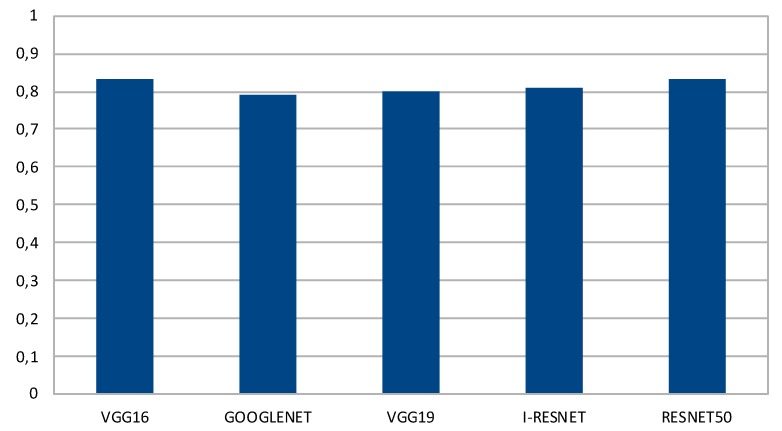
Performance in terms of Deep Convolutional Neural Networks (DCNNs) Accuracy. The best results are achieved by VGG-16 and ResNet50.

As shown above, high values of precision and recall can be achieved, thus demonstrating the effectiveness and the suitability of the proposed approach for classifying the type of user observing a painting. The performance of all DCNNs are shown in Figure 6, in terms of Accuracy. The best results are achieved by VGG-16 and ResNet50 with 83% accuracy.

## 5. Conclusions and Future Works

We described a novel Visual Attentive Model which can recognize museum visitors observing paintings with similar features, chosen by Cultural Heritage experts. The main novelty of the proposal is eye coordinates transformation specifically performed to classify user type. In particular, we have collected eye-tracking data of children and adults looking at five images—the three “Ideal Cities” (Urbino, Baltimore and Berlin), the Inlaid chest in the National Gallery of Marche and Wooden panel in the “Studiolo del Duca” with Marche view. Our described method combines a new coordinates representation from eye sequences by using Geometric Algebra with the deep learning model for automated recognition (to identify, differentiate, or authenticate individuals) of people by the attention focus of distinctive eye movement patterns. We firstly construct an eye-sequence space as a subset of Geometric Algebra to represent the eyes’ coordinates. Then a view transformation is proposed to overcome the diversity of viewpoints.

By comparing DCNNs the approach is able to learn a high level representation of visual attention and to achieve high precision and recall for user classification. The experiments yield high accuracy and demonstrate the effectiveness and suitability of our approach. The exploitation of classification methods for uncovering the user’s behaviours, especially in the CH domain, offers unique advantages for art curators that are interested in enhancing their exhibition. Further investigation will be devoted to improving our approach by employing a larger dataset in order to compare different types of behaviour of users. Moreover, we will extend the evaluation for integrating a mobile eye tracker, as a natural interaction device, into an audio guide system for museum visitors. In fact, eye tracking technology enables new forms of interactions in Augmented and Virtual Reality for creating and exploring virtual spaces with greater ease by navigating the gaze.

## Figures and Tables

**Table 1:** List of the column of the data obtained from the eye tracker.

Column Name	Explanation
*FixationX and FixationY*	is the (X,Y) coordinates of the image where the person, in front of the eye tracker, look on the image. This coordinates are given with reference to the 0 of an image, the top left corner of the image.
*FixationDuration*	is the interval of time that the user focus his attention to the pixel with coordinates (FixationX, FixationY).
*Name*	is the name of the user for the experiment

**Table 2:** Performance evaluation of the DCNNs for the users’ classification.

DCNNs	Users’ Class	Precision	Recall	F1-Score
**VGG-16 [17]**	**Adults**	1.00	0.67	0.80
	**Children**	0.75	1.00	0.86
	***MEAN***	***0.875***	***0.835***	***0.830***
**VGG-19 [17]**	**Adults**	1.00	0.60	0.75
	**Children**	0.72	1.00	0.83
	***MEAN***	***0.860***	***0.800***	***0.790***
**Inception-ResNet [18]**	**Adults**	1.00	0.62	0.77
	**Children**	0.73	1.00	0.84
	***MEAN***	***0.865***	***0.810***	***0.805***
**GoogLeNet [19]**	**Adults**	1.00	0.58	0.74
	**Children**	0.71	1.00	0.83
	***MEAN***	***0.855***	***0.790***	***0.785***
**ResNet-50 [20]**	**Adults**	1.00	0.67	0.80
	**Children**	0.75	1.00	0.86
	***MEAN***	***0.875***	***0.835***	***0.830***
